# *Borrelia burgdorferi* Induces TLR2-Mediated Migration of Activated Dendritic Cells in an *Ex Vivo* Human Skin Model

**DOI:** 10.1371/journal.pone.0164040

**Published:** 2016-10-03

**Authors:** Lauren M. K. Mason, Alex Wagemakers, Cornelis van ‘t Veer, Anneke Oei, Wouter J. van der Pot, Kalam Ahmed, Tom van der Poll, Teunis B. H. Geijtenbeek, Joppe W. R. Hovius

**Affiliations:** 1 Center for Experimental and Molecular Medicine, Academic Medical Center, Amsterdam, the Netherlands; 2 Department of Medical Microbiology, Academic Medical Center, Amsterdam, the Netherlands; 3 Department of Plastic Surgery, Kennemer Gasthuis, Haarlem, the Netherlands; 4 Division of Infectious Diseases, Academic Medical Center, Amsterdam, the Netherlands; 5 Department of Experimental Immunology, Academic Medical Center, Amsterdam, the Netherlands; University of Kentucky College of Medicine, UNITED STATES

## Abstract

*Borrelia burgdorferi* is transmitted into the skin of the host where it encounters and interacts with two dendritic cell (DC) subsets; Langerhans cells (LCs) and dermal DCs (DDCs). These cells recognize pathogens via pattern recognition receptors, mature and migrate out of the skin into draining lymph nodes, where they orchestrate adaptive immune responses. In order to investigate the response of skin DCs during the early immunopathogenesis of Lyme borreliosis, we injected *B*. *burgdorferi* intradermally into full-thickness human skin and studied the migration of DCs out of the skin, the activation profile and phenotype of migrated cells. We found a significant increase in the migration of LCs and DDCs in response to *B*. *burgdorferi*. Notably, migration was prevented by blocking TLR2. DCs migrated from skin inoculated with higher numbers of spirochetes expressed significantly higher levels of CD83 and produced pro-inflammatory cytokines. No difference was observed in the expression of HLA-DR, CD86, CD38, or CCR7. To conclude, we have established an *ex vivo* human skin model to study DC-*B*. *burgdorferi* interactions. Using this model, we have demonstrated that *B*. *burgdorferi*-induced DC migration is mediated by TLR2. Our findings underscore the utility of this model as a valuable tool to study immunity to spirochetal infections.

## Introduction

Lyme borreliosis is caused by *Borrelia burgdorferi* sensu lato, a bacteria which is transmitted into the skin through infected *Ixodes* tick bites [1]. Upon entering the skin the bacteria is recognized by cells of the immune system, such as dendritic cells (DCs). DCs are professional antigen presenting cells (APCs) that patrol barrier sites for invading pathogens, which they phagocytose and process into antigen to present to CD4+ T cells, which they encounter upon migration into draining lymph nodes. Thus, DCs form a crucial bridge between the innate and adaptive immunity [2].

Two subsets of DC reside in the skin; epidermal Langerhans cells (LCs) and dermal dendritic cells (DDCs). These are among the first cells that *B*. *burgdorferi* encounter upon transmission [3]. DCs recognise pathogens through pattern recognition receptors (PRR). TLR2 has been acknowledged as one of the principal PRRs involved in recognition of borrelial lipoproteins such as outer surface protein C (OspC) [4, 5]. Upon recognition, DCs have been shown *in vitro* to phagocytose *B*. *burgdorferi*, produce cytokines and gain a more mature phenotype, characterized by enhanced expression of cell-surface markers such as CD83 and CD86 and the antigen presenting molecule major histocompatibility complex (MHC)-II [6, 7]. LCs containing *B*. *burgdorferi* are found abundantly in erythema migrans lesions [8], however, it is unclear whether they play a role in immunity against *B*. *burgdorferi*, particularly as LCs have a low expression of TLR2 and their contribution to anti-bacterial immunity is uncertain [9, 10].

In order to study the response of both DC subsets and the factors that influence this in a more physiological setting, we established an *ex vivo* model in which *B*. *burgdorferi* is injected into full-thickness human skin to mimic tick bite transmission and studied the migration and maturation of responding DCs.

## Materials and Methods

### Human skin injection model

Human surplus skin was obtained from healthy donors undergoing corrective breast or abdominal surgery at the Kennemer Gasthuis Haarlem, University Medical Center Utrecht and Catharina Hospital Eindhoven, the Netherlands. Skin was used less than 24 hours after removal, and cleansed with 70% alcohol prior to inoculation. A total of 10^2^−10^6^ spirochetes, 100 ng/ml *Escherichia coli* LPS (Invitrogen, Paisley, UK) or 500 ng/ml Pam3CSK4 (Invivogen, San Diego, CA) were injected intradermally in 50 μl PBS using 20 gauge needles (BD, Franklin Lakes, NJ). In some experiments, inocula contained 10 μg/ml αTLR2.5 (generously provided by Loek Willems, HBT, Uden, Netherlands). Eight mm biopsies were taken immediately from the injection site and washed in IMDM (Life Technologies, Paisley, UK) with 2% FCS (Lonza, Verviers, Belgium) before being transferred into 1 ml IMDM with 10% FCS and L-glutamine (Lonza)—without antibiotics—and incubated for 24, 48 or 72 hours at 37°C with 5% CO_2_. The migrated cells and supernatant from three biopsies, taken from separate random locations of the skin, were pooled to constitute one replicate of each condition.

### Monocyte derived dendritic cell (moDC) generation and stimulation

MoDCs were generated from adherent monocytes isolated from the blood of healthy volunteers by density gradient using Ficoll paque (GE-healthcare, Uppsala, Sweden) and Percoll (GE-healthcare) and cultured in Roswell Park Memorial Institute (RPMI) 1640 medium (Gibco, Paisley, UK) with 10% FCS and L-glutamine supplemented with 1000 U/ml interleukin (IL)-4 (Prospec, East Brunswick, NJ, USA) and 50 ng/ml Granulocyte Macrophage Colony-Stimulating Factor (GM-CSF) (Gibco) at 37°C with 5% CO_2_, as previously described [11]. A total of 1 x 10^5^ moDCs in RPMI medium with 10% FCS and L-glutamine were stimulated for 48 hours with different multiplicities of infection of *B*. *burgdorferi*, 10 ng/ml LPS or 50 ng/ml Pam3CSK4.

### *B*. *burgdorferi* culture

*Borrelia burgdorferi* senso stricto strain B31 and wildtype, OspC-deficient and OspC-complemented *B*. *burgdorferi* strain 297 (courtesy of Erol Fikrig, Yale University, New Haven, CT, USA) [12] were cultured in modified Kelly-Pettenkofer medium (AMC, Amsterdam, Netherlands) plus sodium bicarbonate supplemented with 6% rabbit serum (Sigma-Aldrich, Zwijndrecht, Netherlands) [13] at 33°C until cultures reached mid-late log growth phrase. Spirochetes were assessed for motility, quantified by dark-field microscopy and recovered by centrifugation at 4000xg for 8 minutes and resuspended in PBS (Fresenius Kabi, Graz, Austria) (*ex vivo* experiments) or RPMI (*in vitro* experiments). Spirochetes were passaged no more than 5 times. To test spirochetes viability 72 hours post-inoculation, 10 μl biopsy medium was cultured in 7 ml modified Kelly-Pettenkofer medium and inspected by dark-field microscopy.

### Cell Analysis and flow cytometry

Cells were harvested, washed twice, counted and resuspended in FACS buffer (0.5% BSA, 0.01% NaN_3_ and 0.35 mM EDTA in PBS). Cells were stained with fluorophore-conjugated antibodies against HLA-DR, CD11c, CD83, CD86, CD1a, CCR7, CD38, CD68 (BD) or Langerin (Beckman Coulter, Brea, CA) and flow-cytometry was performed using a FACS-CANTO II (BD). The results were analysed using Flow-Jo (Treestar, Ashland, OR) and Prism5 (GraphPad Software, La Jolla, CA).

### Cytokine measurement

Concentrations of the cytokines IL-1β, IL-6, IL-8, TNFα, IL-12p70 and IL-10 were measured in biopsy supernatant using a human inflammatory cytometric bead array (CBA) kit (BD), according to the manufacturer’s instructions. The detection range was 10 pg/ml– 1 μg/ml. Results were analysed using Flow-Jo and Prism5.

### Statistics

Statistical analysis was performed on each set of results with Prism5, using a Kruskal-Wallis test for overall statistical significance. Differences between conditions were analysed posthoc using Dunn’s post test. Data was considered significant at *p*<0.05, with one asterisk (*) representing 0.01<*p*<0.05, two asterisks (**) representing 0.001<*p*<0.01 and three asterisks (***) representing 0.0001<*p*<0.001.

### Ethics statement

The Medical Ethical Committee of the Academic Medical Center, Amsterdam, deemed that no approval was necessary for the use of (otherwise) discarded, anonymous tissue. *In vitro* experiments were performed using surplus healthy volunteer blood collected for the purpose of a separate study. Written informed consent was obtained from human blood donors in accordance with protocols approved by the Medical Ethics Committee of the Academic Medical Center (Approval number NL34294.018.10) and the declaration of Helsinki.

## Results

### *B*. *burgdorferi* interacts with skin DCs and survives in the skin

**Fig 1:**
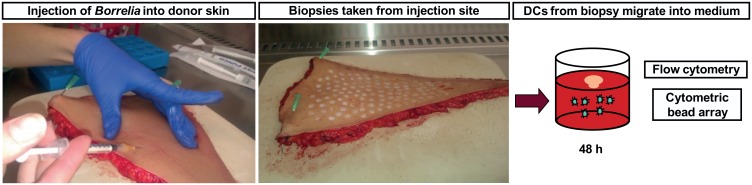
*B*. *burgdorferi* was injected intradermally into the skin and biopsies were taken from the injection site. *Borrelia burgdorferi* strain B31 in PBS was injected intradermally into full-thickness human donor skin (left panel). 8 mm biopsies were taken from around the injection site (middle panel) and then cultured in medium in a 48-wells plate for 48 hours. The migrated cells were collected and analysed using flow cytometry, and a cytometric bead array was performed to measure cytokines in the supernatant (right panel).

*B*. *burgdorferi* was injected intradermally into human skin to mimic transmission via tick bite. Biopsies were taken immediately from around the injection site and cultured in medium for 24–72 hours to allow responding DCs to migrate out of the skin and into the medium (Fig 1). Biopsy-containing medium cultured 72 hours post-inoculation in modified Kelly–Pettenkofer medium revealed the presence of viable spirochetes, confirming that *B*. *burgdorferi* survive in the skin for the duration of the experiment (data not shown).

### DCs migrate out of the skin in response to injection of *B*. *burgdorferi*

**Fig 2:**
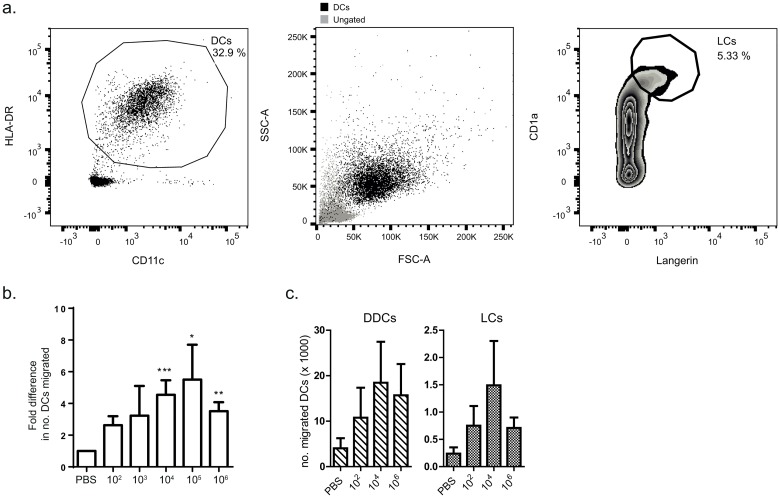
LCs and DDCs migrate out of the skin in response to *B*. *burgdorferi* injection. Flow cytometry was performed on cells collected from biopsy medium. a) DCs were defined as HLA-DR+/CD11c+ cells (left panel). This population is shown in black in the forward/side scatter plot (centre panel). LCs were defined as CD1a+/Langerin+ cells and encompassed approximately 5% of the DC population (right panel). b) To calculate the number of migrated DCs per biopsy, the total number of cells retrieved from biopsy medium was counted (between 5x10^3^ and 2x10^6^ total cells per biopsy) and the percentage of HLA-DR+/CD11c+ cells was taken. Higher doses of *B*. *burgdorferi* induced migration of DCs that was significantly greater than PBS alone at 48 hours. The data was also significant when calculated for actual cell numbers. The graph shown depicts data pooled from 12 independent experiments, ± SEM. c) Both DDCs and LCs migrated in response to *B*. *burgdorferi* injection. This graph represents the pooled data of 4 independent experiments, ± SEM.

Next, the migrated cells were phenotyped and quantified by flow cytometry. DCs were defined as HLA-DR^+^/CD11c^+^ cells and contributed to approximately 50% of total cells. LCs and DDCs were differentiated on the basis of CD1a and Langerin staining [14, 15] (Fig 2a); CD1a^hi^/Langerin^+^ LCs contributed to merely 2–6% of the total DCs. Injection of *B*. *burgdorferi* into the skin induced migration of DCs, as approximately four times as many DCs were collected from biopsies injected with 10^4^−10^6^ compared to PBS injected biopsies at 48 hours after inoculation (Fig 2b). DC migration appeared to peak with inoculation with 10^5^ *B*. *burgdorferi*, as fewer DCs were collected from 10^6^-injected biopsies, however, this difference was not significant. At 24 hours after inoculation, low numbers of migrated DCs were collected. However, this increased and plateaued at 48 hours, as by 72 hours similar numbers of migrated DCs were observed (data not shown). Of the migrated cells, a low percentage was CD1a^hi^ LCs, however, these migrated as readily as DDCs in response to *B*. *burgdorferi* (Fig 2c). CD68 expression of collected cells was also measured to detect the presence of macrophages in the migrated cell population, however no positive cells were detected (data not shown), suggesting that these cells remain in the skin.

### Migrated DCs are mature and express activation markers

**Fig 3:**
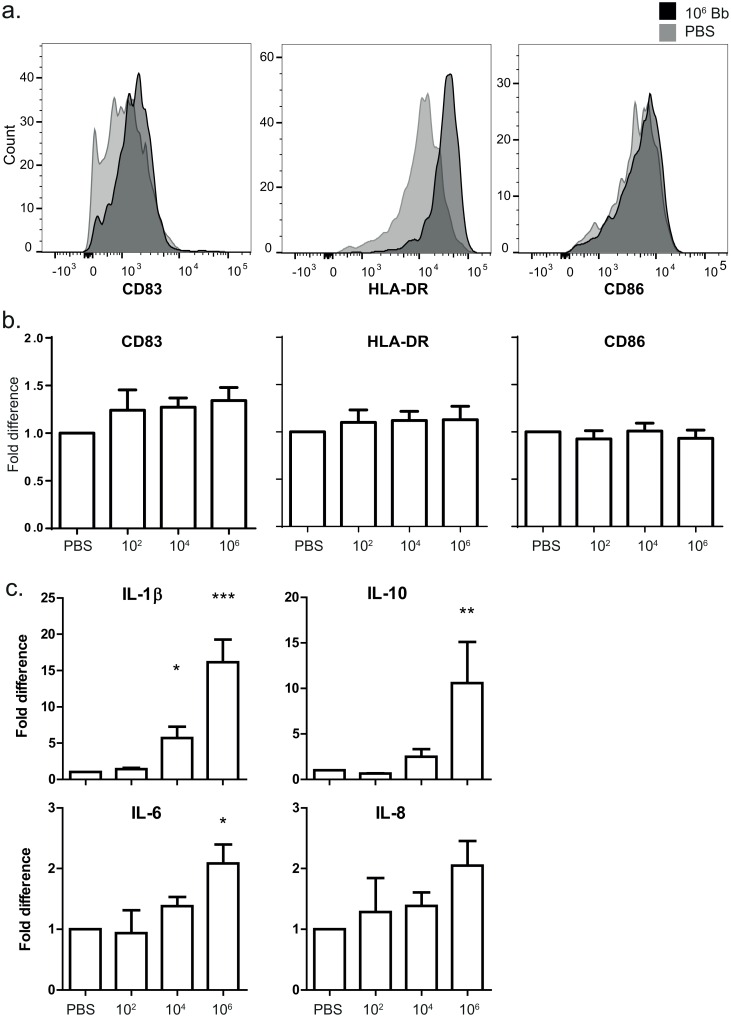
Migrated cells are mature and express activation markers. a) Flow cytometry was performed on cells to measure expression of cell-surface markers CD83, HLA-DR and CD86. The geometric mean fluorescence intensity of CD83 and HLA-DR on DCs from 10^6^ *B*. *burgdorferi*-injected biopsies (dark grey histograms) was modestly raised compared to DCs from PBS-injected biopsies (light grey histograms). CD86 expression was similar in the two conditions. These histograms are based on one donor. b) Expression of these markers varied across donors and there were no significant differences between the groups when data were pooled. These graphs depict the fold difference (MFI stimulus/MFI PBS) and are based on pooled data of 9 independent donors, ± SEM. c) A CBA was used to measure cytokine concentration in biopsy medium. Modestly higher levels of IL-1β, Il-10 and IL-6 were detected in the supernatant of biopsies inoculated with 10^6^ *B*. *burgdorferi* compared to PBS 48 hours after inoculation. These graphs depict the fold difference (pg/ml stimulus/pg/ml PBS) and are based on pooled data of 8 independent donors, ± SEM. Mean concentrations in pg/ml (± SEM) measured in PBS-injected biopsies were as follows: IL-1β: 22.5 (7.5); IL-10: 9.9 (2.8); IL-6: 32250 (12465); IL-8: 29492 (15752). Actual concentrations of IL-1β and IL-10 were also significantly higher in the supernatant of biopsies inoculated with 10^6^ *B*. *burgdorferi* compared to PBS.

In order to assess the phenotype of migrated cells, the expression of cell-surface markers CD83, CD86 and HLA-DR was measured by flow cytometry 48 hours after inoculation. Only CD83 expression was slightly increased in DCs migrated from biopsies injected with 10^6^ *B*. *burgdorferi* compared to PBS-injected biopsies (Fig 3a), however, this appeared to be donor-dependent as a significant increase was seen in 6/10 donors measured, but pooled data showed no significant differences in the expression of activation markers (Fig 3b). Furthermore, we detected no significant differences in the expression of these markers when cells were divided according to CD1a expression (data not shown). Expression of CCR7 and CD38, molecules involved in DC migration [16], similarly did not differ between PBS and *B*. *burgdorferi*-injected biopsies (S1a Fig). To confirm that *B*. *burgdorferi* was unable to induce CCR7 and CD38 expression, we stimulated monocyte-derived DCs (moDCs) with *B*. *burgdorferi*. Indeed, *B*. *burgdorferi* did not induce upregulation of these markers in moDCs either (S1b Fig). In addition to measuring activation markers by flow cytometry, the concentration of pro- and anti-inflammatory cytokines was measured in the biopsy-containing medium. Twenty-four hours after inoculation, cytokine levels were low in all conditions (data not shown), however, at 48 hours significantly higher levels of IL-6, IL-1β and IL-10 were measured in the media of biopsies injected with 10^6^ spirochetes compared to PBS-injected biopsies. Biopsies injected with 10^4^ spirochetes also produced significantly higher levels of IL-1β (Fig 3c). Both IL-12 and TNF-α levels were below the detection limit.

### Migration is TLR2 mediated

**Fig 4:**
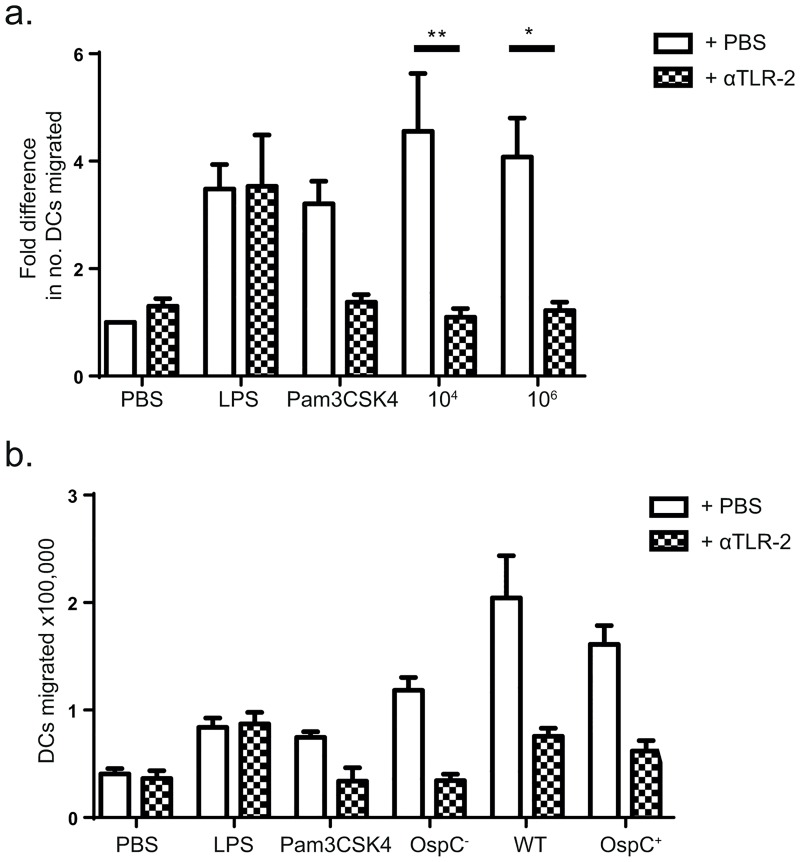
Migration is TLR-2 mediated and OspC independent. a) Addition of a blocking αTLR2-antibody inhibited the *B*. *burgdorferi*-induced increase in migration of DC out of the skin. The increase in DC migration induced by pam3CSK4, but not LPS, was also inhibited by blocking TLR2. The graph shown is based on the pooled data of 7 independent experiments, ± SEM. b) Inoculation of 10^4^ *B*. *burgdorferi* lacking OspC (OspC^-^) induced migration of DC similarly to wildtype (WT) and OspC-complemented *B*. *burgdorferi* (OspC+). This was inhibited by blocking αTLR2 antibodies. Error bars are based on SEM of triplicate measurements from one donor, representative of two independent experiments.

A blocking anti-TLR2 antibody was added to inocula in order to determine whether TLR2 activation is implicated in *B*. *burgdorferi-*induced migration of DCs. Indeed, αTLR2 significantly suppressed the increase in migration of DCs due to *B*. *burgdorferi*, to PBS-injection levels (Fig 4a). Pam3CSK4-mediated migration, but importantly, not LPS-mediated migration, was also inhibited, confirming that the observed effect was a direct effect of blocking TLR2. CD1a^hi^ DC migration in response to *B*. *burgdorferi* also appeared to be inhibited by αTLR2 (data not shown). In contrast, cytokine release was not significantly inhibited by αTLR2 (S2 Fig). The TLR2 agonist OspC is one of the main lipoproteins expressed on the outer surface of *B*. *burgdorferi* when entering the host skin [17]. To investigate whether OspC recognition by TLR2 is of paramount importance for DC migration, an OspC-lacking mutant strain of *B*. *burgdorferi*, wildtype and an OspC-complemented strain of *B*. *burgdorferi* [12] were injected into skin. The OspC-lacking strain induced migration of DCs in a comparable manner to the wildtype and OspC-complemented strains, and was blocked by αTLR2 (Fig 4b), signifying that spirochetal factors other than OspC contribute to migration in a TLR2-dependent manner.

## Discussion

We have established an *ex vivo* human skin model to study the response of DCs to entry of *Borrelia burgdorferi* into the skin. Both LCs and DDCs, migrated out of the skin in response to intradermal injection of *B*. *burgdorferi*. Migrated cells had an activated phenotype and cytokines were produced in the skin in response to *B*. *burgdorferi*. Finally, migration of DCs induced by *B*. *burgdorferi* was TLR2-mediated and OspC-independent.

In this model, we inoculated human skin with 10^2^−10^6^ spirochetes and took 8 mm biopsies. The inoculum used reflects the real-life early stages of *B*. *burgdorferi* infection as these inocula are rather comparable to numbers of spirochetes detected in 2mm biopsies of erythema migrans lesions [18]. In addition, we could cultivate *B*. *burgdorferi* out of biopsies 72 hours after inoculation, indicating that DCs are interacting with viable spirochetes in this model. It is possible that when inoculating the skin with large doses of cultured *B*. *burgdorferi*, some bacteria may die quickly which could potentially affect the way that DCs are stimulated and therefore the readouts generated in this model, as stimulation with live *B*. *burgdorferi* elicits different responses than that of dead or lysed *B*. *burgdorferi* [19]. DCs are professional APCs, which recognize and capture *B*. *burgdorferi*, and migrate out of the skin in response, as we show here. In the skin, two broad classes of DC exist; DDCs, which differentiate in situ from conventional DC progenitors migrated from bone marrow to the skin, and LCs, which are maintained in the epidermis [20]. We observed increased migration of both DC classes out of the skin in response to *B*. *burgdorferi* doses of 10^4^−10^6^, in similar numbers to that of LPS-inoculated skin. Numbers of migrated LCs comprised merely 2–6% of total migrated DCs. This may reflect a slower LC migration rate [21], although the proportions of CD1a^hi^ and CD1a^lo^ DCs recovered remained consistent over time (data not shown). It is likely that intradermal inoculation (as with tick inoculation), bypasses epidermal LCs so that they are less likely to encounter *B*. *burgdorferi* and become activated compared to DDCs. In addition to the broad classification of LCs and DDCs, DDCs may be further classified into various subsets according to the expression of markers including CD14, CD1c and CD141. Specializations and various functions have been attributed to the different subsets, however their relative contributions to the skin immunity is far from clear [22]. The role and the contribution of the different DDC subsets in response to *B*. *burgdorferi* in the present study warrant further investigations.

In order to characterize the phenotype of migrated DCs, we measured the expression of several surface markers that typify DC maturation: CD83, the co-stimulatory molecule CD86, and HLA-DR, the molecule responsible for antigen presentation [2]. As expected, maturation marker expression was increased on the migratory DC population and no differences in HLA-DR and CD86 expression were observed between the various conditions, although significant CD83 upregulation was detected in a subset of donors after inoculation with 10^6^ *B*. *burgdorferi*. This is most likely explained by the fact that in our model those DCs that have migrated—also in response to PBS—have a mature and activated phenotype. Nonetheless, at least for the higher inocula, inoculation of biopsies with *B*. *burgdorferi* also induced production of the pro-inflammatory cytokines IL-6, IL-8, IL-1β and the anti-inflammatory cytokine IL-10. Although we and others have previously shown that *B*. *burgdorferi* induces production of these cytokines in DCs, we cannot rule out that these cytokines are produced by other cells present in the skin, as it has been reported that keratinocytes, recognize and produce IL-8 when stimulated *in vitro* with *B*. *burgdorferi* [23]. Similarly, fibroblasts upregulate IL-8 and IL-6 in response to *B*. *burgdorferi* stimulation [24] and both cell types are capable of producing other cytokines in response to various stimuli [25–27].

TLR-induced CCR7 upregulation and subsequent CCL19 and CCL21 responsiveness is widely acknowledged to be the principal manner by which DCs migrate [16]. The ectoenzyme CD38 is also reported to be a major factor involved in DC migration [28]. In this model, expression of these molecules on DC that migrated in response to *B*. *burgdorferi* was comparable to PBS, although LPS and Pam3CSK4 did induce significant upregulation of CCR7 and CD38, respectively. One explanation for this could be that, as with the maturation marker expression, we are examining migrated cells, which presumably migrated due to high expression of these markers. In order to investigate this hypothesis, we stimulated moDCs with *B*. *burgdorferi in vitro*. Interestingly, *B*. *burgdorferi* did not appear to induce CCR7 or CD38 upregulation. This is in line with similar experiments in which *B*. *garinii* failed to induce upregulation of CCR7 and CD38 expression on moDCs 7, 24 and 48 hours after stimulation [29]. Furthermore, *B*. *garinii*-stimulated DCs did not migrate effectively toward CCL19 and CCL21 *in vitro* and towards lymph nodes in mice compared to DCs stimulated with *Escherichia coli* [30]. Obviously these models differ greatly from our human skin model; measuring the CCR7 and CD38 expression of the pre-migration skin DC population may shed light on the ability of *B*. *burgdorferi* to induce upregulation of these factors and help to determine whether this is the mechanism by which migration takes place in this model. The complex mechanisms behind DC migration have not been completely elucidated [16], but involve factors including prostaglandin E2 and matrix metalloproteinases [31] and are also largely regulated by supporting cells such as keratinocytes and fibroblasts [32]. We show here that *B*. *burgdorferi*-induced DC migration is mediated by TLR2, as blocking the receptor using an antagonistic αTLR2 antibody [33] abrogated the increase in migration. TLR2 recognises diacylated and triacylated lipopeptides by forming heterodimers with TLR1 and TLR6 [34, 35]. The TLR2-mediated recognition of *B*. *burgdorferi* induces the Myeloid differentiation primary response 88-dependent activation of nuclear factor κB signaling cascade, triggering transcription of co-stimulatory molecules, pro-inflammatory and anti-inflammatory cytokines, matrix metalloproteinases and adhesion molecules, which enable the maturation and migration of DCs [3]. Although DDCs recognize a broad range of pathogens, it has been reported that LCs have minimal expression of the TLRs involved in bacterial recognition, including TLR2 [9, 10]. Despite this, we observed that LCs readily migrated out of biopsies in response to *B*. *burgdorferi* and that this was also mediated through TLR2. This may be caused by the activation of the surrounding skin cells, since keratinocytes and fibroblasts express TLR2, recognize *B*. *burgdorferi* and are known to communicate with and play a vital supporting role in skin DC immunology [24, 36, 37]. Alternatively, TLR2 expression may be increased on LCs upon stimulation, as increased TLR2 expression has been described in CD11^+^ DCs in skin infiltrates after intradermal injection of a synthetic analog of OspC, although the investigators did not differentiate between LCs and DDCs [38]. Furthermore, LCs isolated from epidermis upregulated maturation markers in response to TLR2 agonists despite weak TLR2 expression [9]. Cytokine production was not significantly altered in biopsies injected with αTLR2. Apart from TLR2, *B*. *burgdorferi* has been demonstrated to stimulate other PRR such as TLR5, 7, 8 and 9 [3] which may account for the discrepancy. Although OspC, a *B*. *burgdorferi* lipoprotein that is extensively expressed during early mammalian infection in the skin, is undoubtedly implicated in the recognition of *B*. *burgdorferi* by DCs, we have shown that it is not crucial to the TLR2-mediated migration in this model.

In conclusion, we have established an *ex vivo* human skin model in which we can study the migration and activation of DC subsets and cytokine production in response to *B*. *burgdorferi*. Using this model, we have demonstrated that migration is TLR2-dependent and OspC-independent. This model might prove to be a suitable tool to investigate factors that affect *B*. *burgdorferi*-DC interactions, such as components of tick saliva [3, 39]. Indeed, preliminary experiments demonstrate that salivary gland extract inhibits *B*. *burgdorferi*-induced migration of DCs in this model (data not shown). In addition, this model could be extrapolated to other spirochetal agents. The present study demonstrates that using an *ex vivo* skin model is a valid manner in which to explore DC interactions with bacteria while maintaining the skin DCs in their complex tissue microenvironment. Crucially, it also provides a viable alternative to the use of animal models.

## Supporting Information

S1 Fig *B*. *burgdorferi* did not induce upregulation of migration factors CCR7 and CD38. a) Cell surface markers CCR7 and CD38 were measured on DCs migrated out of biopsies by flow cytometry 48 hours after inoculation. Although LPS induced increased expression of CCR7, and Pam3CSK4 induced increased expression of CD38, the expression of these markers on DCs from *B*. *burgdorferi*-inoculated biopsies was comparable to that of PBS. Error bars are based on SEM of triplicate measurements from one donor, representative of three independent experiments. b) CCR7 and CD38 expression was also measured on moDCs stimulated with *B*. *burgdorferi* at an MOI of 10 or 100 for 48 hours. Again, expression of these markers after *B*. *burgdorferi*-stimulation was comparable to PBS, while LPS induced a highly significant increase in expression. Error bars are based on SEM of triplicate measurements from one donor, representative of two independent experiments.
(EPS)

S2 Fig Blocking TLR2 does not abrogate *B*. burgdorferi-induced cytokine production. Cytokine concentration in biopsy medium was measured by CBA. The concentration of cytokines measured in the media of biopsies in which a blocking α-TLR2 antibody was added to the *B*. *burgdorferi* inoculum was not significantly different to biopsies inoculated with *B*. *burgdorferi* alone. These graphs depict pooled data of 6 independent donors, ± SEM.
(EPS)
